# P-486. Implementation of a Proactive and Standardized Approach to Isolation Discontinuation and Survey of Outcomes (IP-SAID-SO)

**DOI:** 10.1093/ofid/ofaf695.701

**Published:** 2026-01-11

**Authors:** Kelsey L Rowe, Patrick J Reich, Stephanie A Fritz, Sara Malone, Carrington Dehart, Ashley Lloyd, Geoffrey Ikpeama, Emily J Jacoby, Emily Hunter, Louise Jadwisiak

**Affiliations:** Washington University School of Medicine, Saint Louis, MO; Washington University School of Medicine, Saint Louis, MO; Washington University School of Medicine, Saint Louis, MO; Washington University School of Medicine, Saint Louis, MO; BJC Healthcare, St. Louis, Missouri; St. Louis Children's Hospital, Saint Louis, Missouri; St. Louis Children's Hospital, Saint Louis, Missouri; St. Louis Children's Hospital, Saint Louis, Missouri; BJC, Saint Louis, Missouri; St. Louis Children's Hospital, Saint Louis, Missouri

## Abstract

**Background:**

Isolation precautions protect healthcare workers (HCWs) and patients, but can have significant environmental impacts, high cost, and be a barrier to patient safety and satisfaction. Respiratory viral infections are the most common isolation indication at St. Louis Children’s Hospital (SLCH). However, isolation discontinuation guidelines for respiratory viral infections are poorly defined. This project evaluated a standardized, proactive process for respiratory viral isolation discontinuation.

**Figure:**
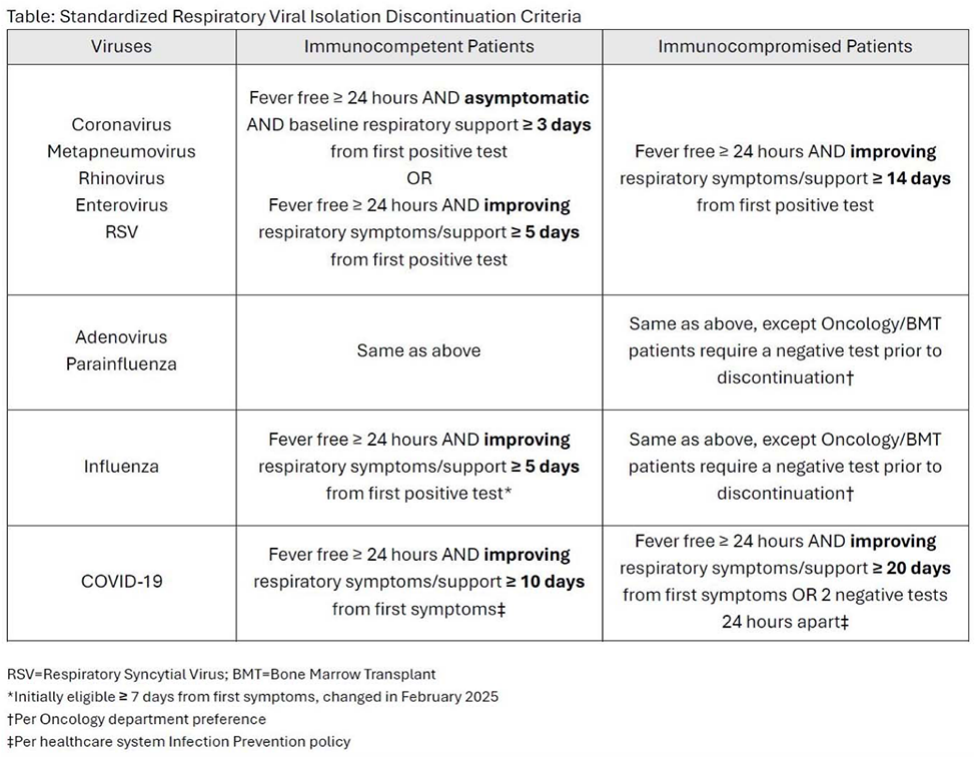

**Figure:**
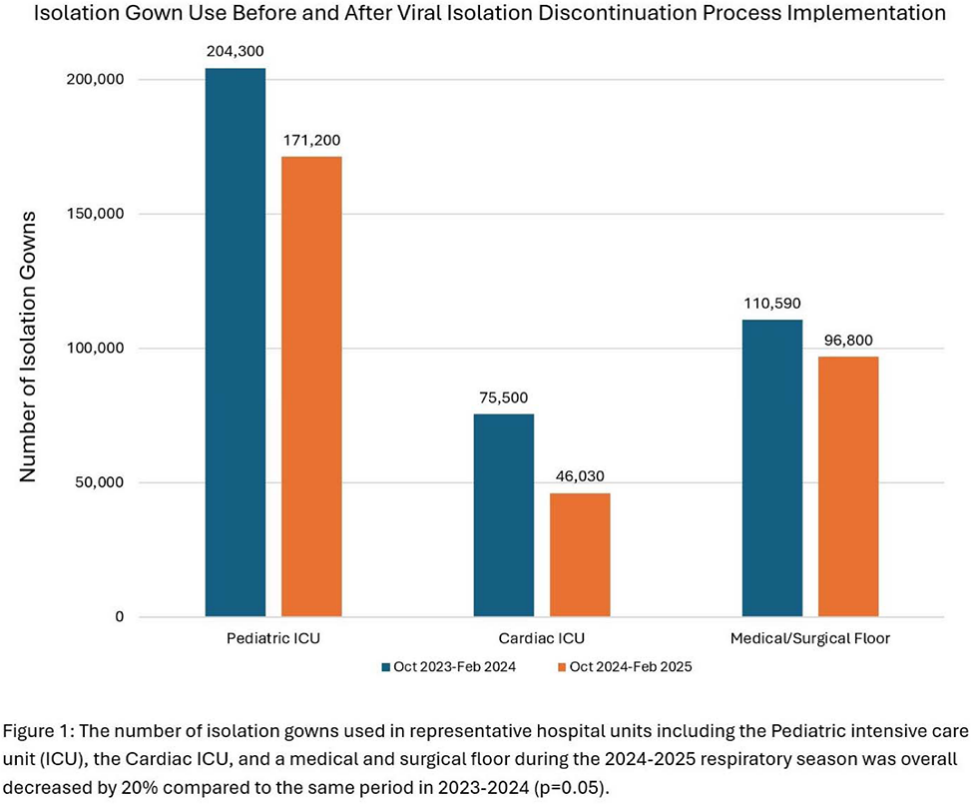

**Methods:**

Hospital-wide isolation discontinuation criteria were standardized for patients with respiratory viral infections (Table). An Electronic Medical Record (EMR) report was created to identify eligible patients. After a pilot in September 2024, the report was run daily from October 2024 through March 2025. Non-intensive care unit (ICU) nosocomial viral infections during this period were reviewed, and monthly rates were compared to the same period in 2023 through 2024 via t-test. Gown usage was also compared via t-test for the Pediatric ICU, Cardiac ICU, and one combined medical and surgical floor. A survey of HCWs assessing this process’s acceptability, appropriateness, and feasibility was conducted via validated measures.

**Figure:**
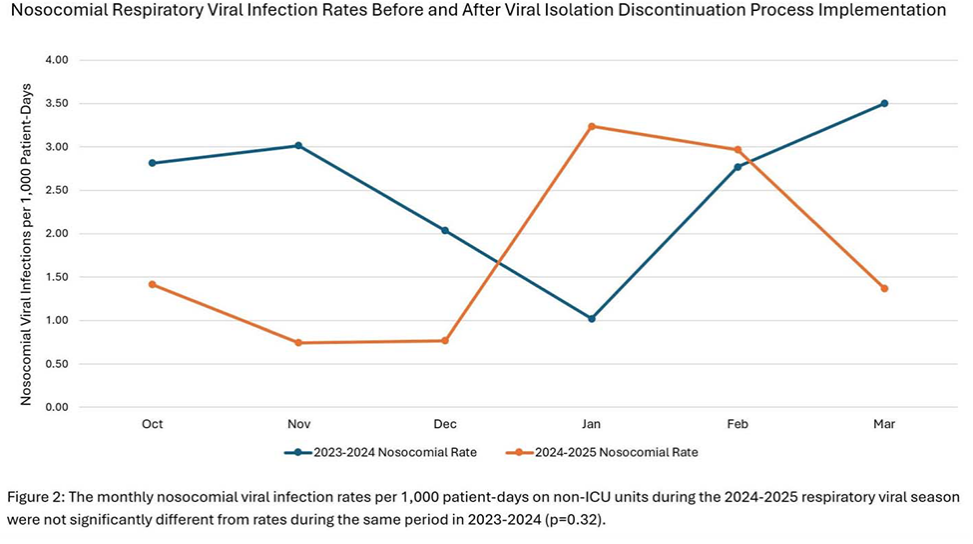

**Figure:**
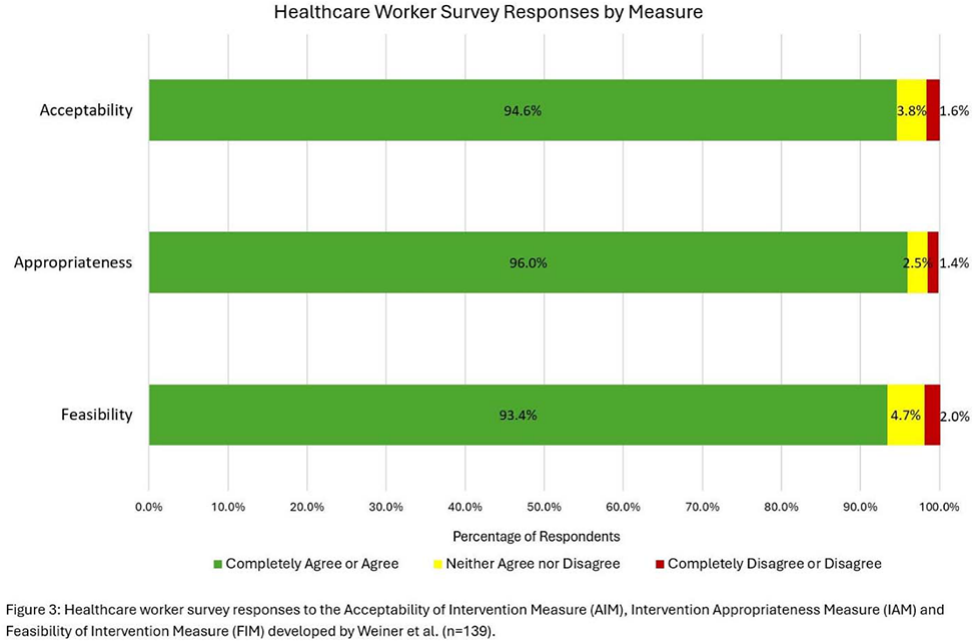

**Results:**

The report identified 1,862 isolation episodes across 873 patients. Adjustments were required for 474 isolations (25%), including 367 over-isolated patients, 103 under-isolated patients, and 4 patients requiring multiple adjustments. Gown usage for the Pediatric ICU, Cardiac ICU, and medical and surgical floor was reduced by 20% (p=0.05) (Figure 1). There was no significant difference in non-ICU nosocomial viral infection rates (p=0.32) (Figure 2). Most HCWs agreed or completely agreed with statements that the isolation removal process is acceptable, appropriate, and feasible (Figure 3).

**Conclusion:**

New standardized respiratory viral isolation discontinuation criteria were successfully implemented using an EMR tool, resulting in decreased gown usage without increasing nosocomial viral infections. Favorable HCW survey responses provided a useful indicator for sustainable implementation. Future directions include EMR report automation to increase the generalizability of this approach.

**Disclosures:**

All Authors: No reported disclosures

